# TMEM16C is involved in thermoregulation and protects rodent pups from febrile seizures

**DOI:** 10.1073/pnas.2023342118

**Published:** 2021-05-10

**Authors:** Tongfei A. Wang, Chao Chen, Fen Huang, Shengjie Feng, Jason Tien, João M. Braz, Allan I. Basbaum, Yuh Nung Jan, Lily Yeh Jan

**Affiliations:** ^a^Department of Physiology, University of California, San Francisco, CA 94158;; ^b^Department of Biochemistry and Biophysics, University of California, San Francisco, CA 94158;; ^c^Department of Anatomy, University of California, San Francisco, CA 94158;; ^d^HHMI, University of California, San Francisco, CA 94158

**Keywords:** TMEM16C (Ano3), febrile seizures, thermoregulation, preoptic area (POA), temperature-sensitive neurons

## Abstract

Febrile seizures (FSs) are the most common convulsion in infancy and childhood. Considering the limitations of current treatments, it is important to examine the mechanistic cause of FSs. Prompted by a genome-wide association study identifying TMEM16C (also known as ANO3) as a risk factor of FSs, we showed previously that loss of TMEM16C function causes hippocampal neuronal hyperexcitability [Feenstra *et al.*, Nat. Genet. 46, 1274–1282 (2014)]. Our previous study further revealed a reduction in the number of warm-sensitive neurons that increase their action potential firing rate with rising temperature of the brain region harboring these hypothalamic neurons. Whereas central neuronal hyperexcitability has been implicated in FSs, it is unclear whether the maximal temperature reached during fever or the rate of body temperature rise affects FSs. Here we report that mutant rodent pups with TMEM16C eliminated from all or a subset of their central neurons serve as FS models with deficient thermoregulation. *Tmem16c* knockout (KO) rat pups at postnatal day 10 (P10) are more susceptible to hyperthermia-induced seizures. Moreover, they display a more rapid rise of body temperature upon heat exposure. In addition, conditional knockout (cKO) mouse pups (P11) with TMEM16C deletion from the brain display greater susceptibility of hyperthermia-induced seizures as well as deficiency in thermoregulation. We also found similar phenotypes in P11 cKO mouse pups with TMEM16C deletion from *Ptgds*-expressing cells, including temperature-sensitive neurons in the preoptic area (POA) of the anterior hypothalamus, the brain region that controls body temperature. These findings suggest that homeostatic thermoregulation plays an important role in FSs.

## Significance

As the most common convulsive disorder in infancy and childhood, affecting 2 to 5% of American children in their first 5 y of life, febrile seizures (FSs) are associated with genetic risk factors, including the *Tmem16c* (*Ano3*) gene. Whereas central neuronal hyperexcitability has been implicated in FSs, whether FSs may result from compromised body temperature regulation is unknown. To approach this question, we developed rodent models of FSs associated with deficient thermoregulation, including conditional knockout mice with TMEM16C eliminated from a hypothalamic neuronal population important for maintaining body temperature but not from most of the cortical and hippocampal neurons and sensory neurons. Our findings raise the possibility that impaired homeostatic thermoregulation could contribute to the risk of FSs.

Body temperature is a critical index of health in mammals, while fever is an evolutionarily conserved response to infection (1). Febrile seizures (FSs), a convulsion triggered by fever in infants and young children ranging from 6 mo to 5 y of age (2–4), are the most common type of convulsion in youth. From 2 to 5% of American children display FSs in their first 5 y of life. Of those who had a first febrile seizure, 40% experienced recurrences of seizures (5). Whether FS genesis is associated with the rate of the core temperature (T_c_) increase and/or when the T_c_ crosses a certain threshold for FS onset is unclear (6, 7). This question is difficult to address in clinical studies because of the paucity of information regarding the rate of T_c_ rise prior to a febrile seizure and the T_c_ at the onset of seizures. Genetic animal models that simulate the syndrome of FSs will greatly facilitate our understanding of FS susceptibility, beyond the known involvement of central neuronal hyperexcitability (8–12). A panoramic view of the pathogenesis of FSs enables future efforts to develop preventive strategies and potential treatments beyond currently available options (13, 14).

A genome-wide association study (GWAS) of Danish children with or without a history of FSs in their first 2 y of life identified four loci as genetic risk factors, with *TMEM16C* (*ANO3*) showing the most significant association with greater FS susceptibility (15). Whereas this variant within intron 1 of *TMEM16C* (15) nearly doubles the risk for febrile seizures (3, 16, 17), whether and how *TMEM16C* gene activity may be involved in febrile seizures remains an open question. TMEM16C belongs to the TMEM16 family that includes Ca^2+^-activated Cl^−^ channels and Ca^2+^-activated scramblases (18, 19). In rat dorsal root ganglia (DRG), TMEM16C enhances the activity of a Na^+^-activated K^+^ channel thereby modulating pain sensitivity (20). Moreover, TMEM16C mutations are found in patients with dominant craniocervical dystonia, with their fibroblasts displaying a reduction in the thapsigargin-sensitive Ca^2+^ pool in the endoplasmic reticulum (21). Multiple missense mutations of *TMEM16C* have been associated with dominant dystonia (22).

To examine the contribution of TMEM16C to FS susceptibility, we tested rat pups with or without TMEM16C (20) for their susceptibility to hyperthermia-induced seizures, an experimental model that simulates febrile seizures (23, 24). We also generated conditional knockout (cKO) mice bearing floxed alleles of *Tmem16c* (*Ano3*) for the purpose of removing TMEM16C via Cre recombinase. By conducting assessment of seizure-like behavior concurrent with T_c_ monitoring of rodent pups upon exposure to heat, we quantified the extent of T_c_ rise leading to the onset of seizures, thereby revealing that mutant pups exhibited greater susceptibility to hyperthermia-induced seizures as well as more rapid increases in T_c_ upon heat exposure.

In mammals, thermoregulation is mediated by the preoptic area of the anterior hypothalamus (POA), involving “temperature-sensitive neurons” in this region that respond to brain temperature change with alteration of spontaneous action potential firing frequency (25–29). Intrigued by the drastic reduction of warm-sensitive POA neurons in rat pups lacking TMEM16C compared to littermate wild-type (WT) controls (15), we identified the *Ptgds* gene as a marker for temperature-sensitive POA neurons (30). Using the mouse line of PGDS-Cre (31), we examined cKO mouse pups with TMEM16C eliminated from temperature-sensitive POA neurons. Our findings that these cKO mutant pups showed not only deficient thermoregulation but also greater susceptibility to hyperthermia-induced seizures raise the possibility that compromised thermoregulation contributes to FS genesis.

## Results

### Rat Pups Without TMEM16C Are More Susceptible to Hyperthermia-Induced Seizures.

**Fig. 1.**
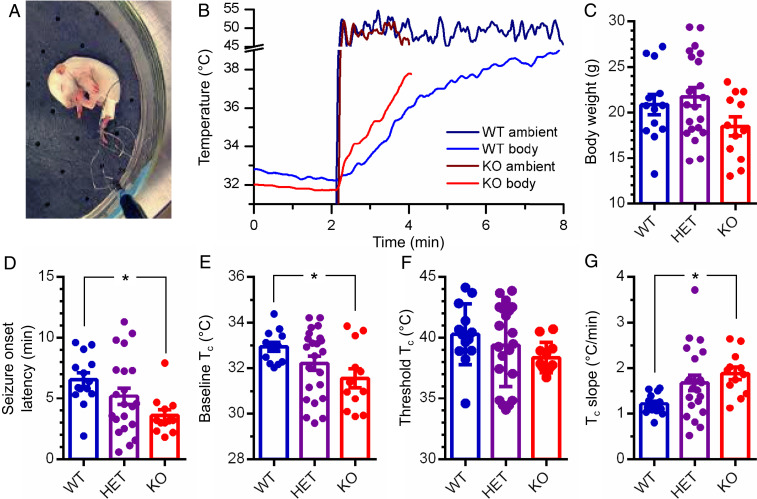
Rat pups without TMEM16C are more susceptible to hyperthermia-induced seizures. (*A*) Photo of a rat pup at the onset of hyperthermia-induced seizure, exhibiting stereotyped behavior of tonic flexion and chewing the hindlimb. (*B*) Sample traces of rat pups’ rectal temperature recording during seizure induction (navy, ambient temperature of wild-type pup; blue, rectal temperature of wild-type pup; brown, ambient temperature of knockout pup; red, rectal temperature of knockout pup). (*C*–*G*) Body weight (*C*), seizure onset latency (*D*), rectal temperature at rest (*E*), rectal temperature at the onset of seizure (*F*), and the slope of rectal temperature change in exposure to heat (*G*) in rat pups with or without TMEM16C (blue, WT, *n* = 14; purple, heterozygous, *n* = 21; red, KO, *n* = 12); *P* > 0.05, one-way ANOVA in *C* and *F*; *P* < 0.05, one-way ANOVA in *D*, *E*, and *G*; *P < 0.05, Tukey’s multiple comparisons test; data are presented as mean ± SEM.

To test *Tmem16c* knockout (KO) rat pups (postnatal day 10 [P10]) for susceptibility to hyperthermia-induced seizures with an established protocol (23, 24), we exposed P10 rat pups to heated air (45 to 50 °C) from a hair dryer, one at a time (Fig. 1). At the onset of hyperthermia-induced seizure, pups suddenly stopped moving, displayed tonic flexion, and chewed their hind limb (Fig. 1*A*). When they were removed from the test chamber and brought to room temperature, pups recovered immediately from the tonic posture and rigidity. Rat pups without TMEM16C (KO, *n* = 12) took less time to develop seizures, compared with their WT (*n* = 14) or heterozygous (HET, *n* = 21) littermates (Fig. 1*D*; *P* < 0.05, one-way ANOVA followed by Tukey’s multiple comparisons test). The body weights of pups with or without TMEM16C were comparable, indicating no growth delay of *Tmem16c* KO rat pups (Fig. 1*C*, *P* > 0.05, one-way ANOVA).

During the test, the pups’ rectal temperature was recorded continuously (Fig. 1*B*). While rat pups without TMEM16C exhibited a lower T_c_ at rest (Fig. 1 *B* and *E*, *P* < 0.05, one-way ANOVA followed by Tukey’s multiple comparisons test), heat exposure caused their T_c_ to increase more rapidly than WT or HET littermate controls (Fig. 1 *B* and *G*, *P* < 0.05, one-way ANOVA followed by Tukey’s multiple comparisons test). The threshold T_c_ at the onset of seizures was comparable for pups with or without TMEM16C (Fig. 1*F*, *P* > 0.05, one-way ANOVA). These results support the hypothesis that a rapid rise in T_c_, other than an altered threshold temperature for seizure onset, contributes to the vulnerability of *Tmem16c* KO rat pups to hyperthermia-induced seizures.

### Mouse Pups with TMEM16C Eliminated from the Brain Exhibit Greater Susceptibility to Hyperthermia-Induced Seizures Associated with Deficient Thermoregulation.

To examine the possible involvement of TMEM16C in different cell types in hyperthermia-induced seizures, we generated a mouse line with floxed *Tmem16c* alleles (*SI Appendix*, Fig. S1 *A* and *B*), for cKO of TMEM16C, to eliminate TMEM16C from central neurons in the brain via Nestin-Cre (*SI Appendix*, Fig. S1*C*, *n* = 4, *P* < 0.01, Student’s *t* test) or from *Ptgds*-expressing cells via PGDS-Cre (31) (*SI Appendix*, Fig. S2).

We first tested cKO mouse pups with TMEM16C removed from central neurons throughout the brain via Nestin-Cre. Upon exposure to rising ambient temperature to elevate T_c_, mouse pups (P11) exhibited stereotyped seizure behaviors progressively in four phases: phase I) hyperactivity, jumping, or rearing; phase II) sudden immobility, ataxia, or jerky gait; phase III) running in circles, whole-body shaking, contractions of hindlimbs and forelimbs with reduced consciousness; and phase IV) tonic convulsions with loss of consciousness (23, 24). Similar to TMEM16C KO rat pups, cKO mouse pups with TMEM16C removed from central neurons in the brain were more susceptible to hyperthermia-induced seizure (*SI Appendix*, Fig. S3 *A*–*D*; *n* = 12 in each genotype; *P* < 0.05 in phases II and III, *P* < 0.01 in phase IV, one-way ANOVA followed by Tukey’s multiple comparisons test). Notably, these cKO mouse pups reached phase IV seizures at lower T_c_ than their WT littermate controls (*SI Appendix*, Fig. S3*E*, *P* < 0.05, one-way ANOVA followed by Tukey’s multiple comparisons test), and heat exposure caused their T_c_ to increase more rapidly than WT littermate controls (*SI Appendix*, Fig. S3*F*, *P* < 0.05, one-way ANOVA followed by Tukey’s multiple comparisons test). These differences cannot be attributed to delayed growth of *Tmem16c* cKO mouse pups, because their body weight was comparable to that of their WT or HET littermates (*SI Appendix*, Fig. S3*G*, *P* > 0.05, one-way ANOVA).

To further assess thermoregulation of the mouse pups, we brought them one at a time from their nests to a rodent incubator with programmable temperature control, in which each pup was exposed to ambient temperatures of 22 °C, 30 °C, and 37 °C for ∼30 min each, and then back to 22 °C (*SI Appendix*, Fig. S4*A*). Note that merely raising the ambient temperature to 37 °C was not sufficient to induce seizures in pups; rather the purpose of this experiment was to examine thermoregulation of mouse pups without seizure onset. The T_c_ of each mouse pup was recorded throughout the test. Mutant mouse pups (P11) with TMEM16C eleminated from the brain exhibited significantly lower T_c_ at ambient temperature of 22 °C or 30 °C, as compared to their WT and HET littermates (*SI Appendix*, Fig. S4 *A*–*D*; WT, *n* = 12; HET, *n* = 12; KO, *n* = 11; *P* < 0.05 in 22 °C ambient temperature, *P* < 0.01 in 30 °C ambient temperature, one-way ANOVA followed by Tukey’s multiple comparisons test), while the T_c_ of mouse pups with the ambient temperature held at 37 °C and during the following recovery period at 22 °C showed no significant difference across different genotypes (*SI Appendix*, Fig. S4 *E* and *F*, *P* > 0.05, one-way ANOVA). As evident from the slope of T_c_ change in response to ambient temperature alteration, *Tmem16c* cKO mouse pups experienced a more rapid increase of T_c_ than their WT littermate controls when the ambient temperature was raised from 30 °C to 37 °C (*SI Appendix*, Fig. S4 *B* and *G*–*J*, *P* < 0.01, one-way ANOVA followed by Tukey’s multiple comparisons test). These findings provide evidence that the neuronal TMEM16C is important for thermoregulation and the propensity for hyperthermia-induced seizure.

### Elimination of TMEM16C from Temperature-Sensitive POA Neurons Causes Deficient Thermoregulation and Increases Hyperthermia-Induced Seizure Susceptibility.

**Fig. 2.**
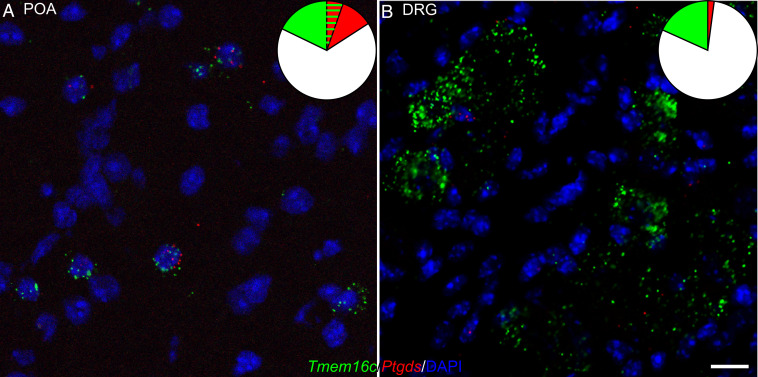
TMEM16C expressed in *Ptgds*-expressing neurons in POA but not in the DRG. (*A* and *B*) In situ hybridization of *Tmem16c* (green), *Ptgds* (red), and DAPI (blue) in POA (*A*) and DRG (*B*) from mouse pups of P11 (three replicates) (Scale bar: 20 µm.)

**Fig. 3.**
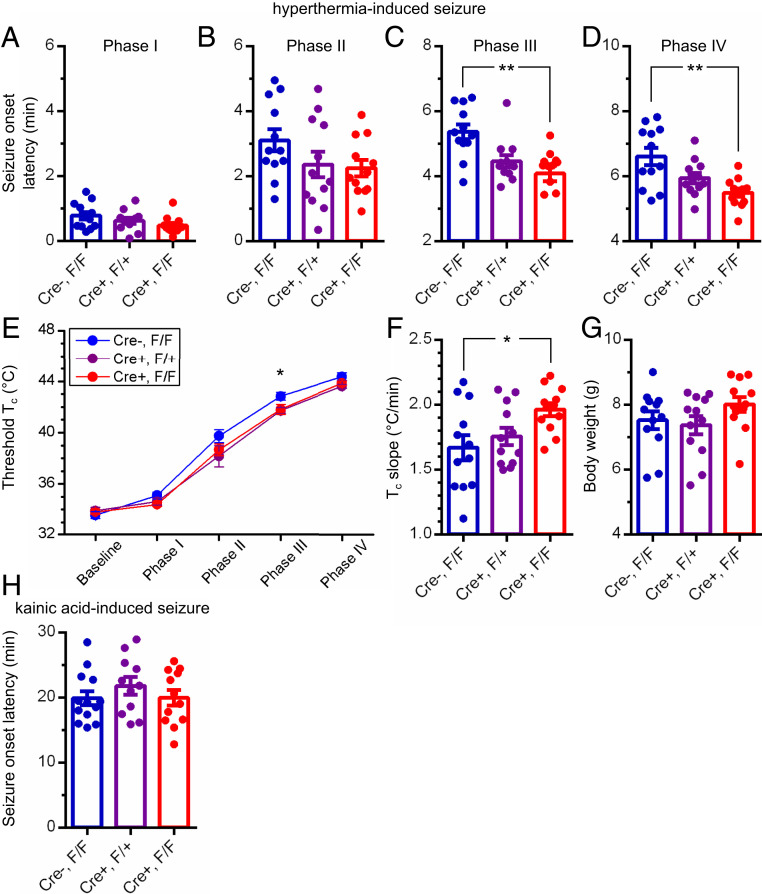
Mouse pups (P11) with TMEM16C removed from *Ptgds*-expressing neurons are more susceptible to hyperthermia-induced seizure. (*A*–*D*) Latency for mouse pups (blue, Cre^−^, flox/flox; purple, Cre^+^, flox/+; red, Cre^+^, flox/flox; *n* = 12 in each genotype) to enter the seizure phases I (*A*, hyperactivity, jumping, or rearing), II (*B*, sudden immobility, ataxia, or jerky gait), III (*C*, circle running, whole-body shaking, contractions of hind- and forelimbs with reduced consciousness, *P* < 0.01, one-way ANOVA), and IV (*D*, tonic convulsions with loss of consciousness, *P* < 0.01, one-way ANOVA); **P < 0.01, Tukey’s multiple comparisons test; data are presented as mean ± SEM. (*E*) Rectal temperature of mouse pups at each phase of a seizure; *P* < 0.05, one-way ANOVA; *P < 0.05, Tukey’s multiple comparisons test in phase III; data are presented as mean ± SEM. (*F* and *G*) Rectal temperature increase rate (*F*, *P* < 0.05, one-way ANOVA; *P < 0.05, Tukey’s multiple comparisons test) and body weight (*G*, *P* > 0.05, one-way ANOVA) of mouse pups in hyperthermia-induced seizure test; data are presented as mean ± SEM. (*H*) Latency for mouse pups to enter a phase IV seizure induced by kainic acid (5 mg/kg, i.p. injection; blue, Cre^−^, flox/flox, *n* = 13; purple, Cre^+^, flox/+, *n* = 11; red, Cre^+^, flox/flox, *n* = 12; *P* > 0.05, one-way ANOVA; data are presented as mean ± SEM).

Having found that temperature-sensitive POA neurons that express *Ptgds* are involved in thermoregulation (30), we wondered whether eliminating TMEM16C from *Ptgds*-expressing cells would affect thermoregulation of mouse pups at an age susceptible to hyperthermia-induced seizure. RNAscope confirmed that *Tmem16c* mRNA was expressed in *Ptgds*-expressing POA neurons (Figs. 2 and 3, replicates) of P11 mouse pups. In their hippocampus and cortex, *Tmem16c* was expressed in a small fraction of the *Ptgds*-expressing neurons (*SI Appendix*, Figs. S2 and S3, replicates). Likewise, there was little overlap between the *Tmem16c* expression and the *Ptdgs* expression in their DRG (Figs. 2 and 3, replicates). We thus used PGDS-Cre to knock out TMEM16C from *Ptgds*-expressing cells, including temperature-sensitive POA neurons, without affecting the integrity of the majority of hippocampal, cortical, and DRG neurons.

Remarkably, these P11 cKO mouse pups displayed greater susceptibility to hyperthermia-induced seizures (Fig. 3 *A*–*D*; *n* = 12 in each genotype; phases III and IV, *P* < 0.01, one-way ANOVA followed by Tukey’s multiple comparisons test). They also exhibited a more rapid T_c_ increase upon heat exposure (Fig. 3*F*, *P* < 0.05, one-way ANOVA followed by Tukey’s multiple comparisons test). By contrast, P11 cKO pups with TMEM16C eliminated from *Ptgds*-expressing cells were comparable to their WT and HET littermates in the susceptibility to chemoconvulsant-induced seizures (Fig. 3*H*; kainic acid intraperitoneal [i.p.] injection, 5 mg/kg; WT, *n* = 13; HET, *n* = 11; KO, *n* = 12; *P* > 0.05, one-way ANOVA). Because of the specificity of the temperature-induced increase in seizure susceptibility, we wondered whether the enhanced susceptibility to hyperthermia-induced seizures might reflect changes in thermoregulation, per se.

**Fig. 4.**
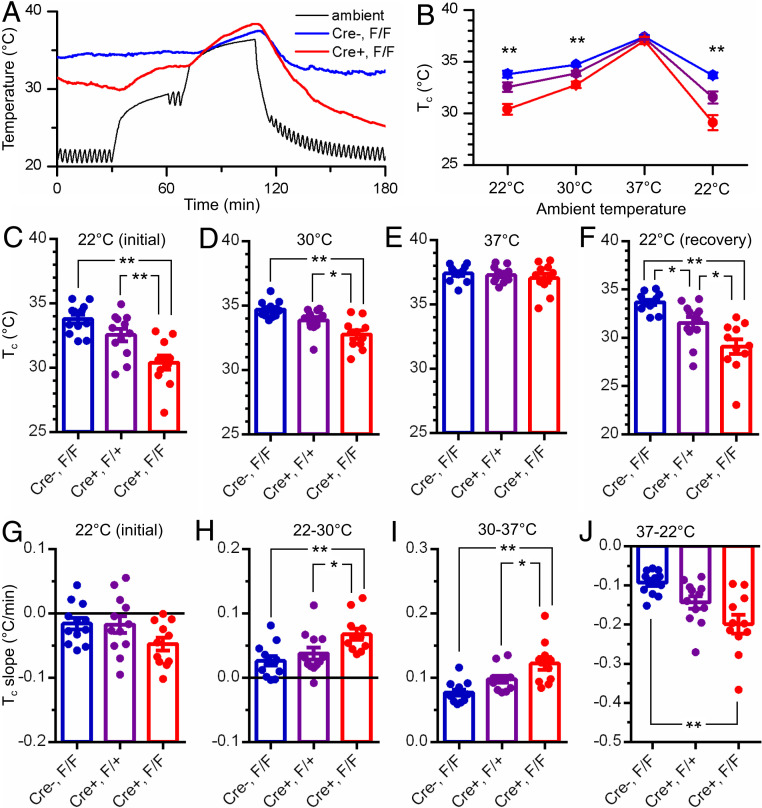
Mouse pups (P11) without TMEM16C in *Ptgds*-expressing neurons have deficiency in thermoregulation. (*A*) Sample traces of rectal temperature recording in mouse pups with or without TMEM16C in *Ptgds*-expressing neurons (blue, Cre^−^, flox/flox, *n* = 12; red, Cre^+^, flox/flox, *n* = 11) in response to elevated ambient temperature (black). (*B*–*F*) Summary of animals’ rectal temperature in test ambient temperatures of 22 °C (*C*, at rest, *P* < 0.01, one-way ANOVA), 30 °C (*D*, *P* < 0.01, one-way ANOVA), 37 °C (*E*), and 22 °C (*F*, recovery from heat exposure, *P* < 0.01, one-way ANOVA); purple, Cre^+^, flox/+, *n* = 12; **P < 0.01; *P < 0.05, Tukey’s multiple comparisons test; data are presented as mean ± SEM. (*G*–*J*) Animals’ rectal temperature change slopes in ambient temperature of 22 °C at rest (*G*), from 22 °C to 30 °C (*H*, *P* < 0.01, one-way ANOVA), from 30 °C to 37 °C (*I*, *P* < 0.01, one-way ANOVA), and from 37 to 22 °C (*J*, recovery from heat exposure, *P* < 0.01, one-way ANOVA); **P < 0.01; *P < 0.05, Tukey’s multiple comparisons test; data are presented as mean ± SEM.

Indeed, P11 cKO mouse pups with TMEM16C removed from *Ptgds*-expressing neurons, when tested individually in a thermal-controlled incubator, exhibited lowered T_c_ when the ambient temperature was held at 22 °C or 30 °C (Fig. 4 *A*–*D*; WT, *n* = 12; HET, *n* = 12; KO, *n* = 11; *P* < 0.01, one-way ANOVA followed by Tukey’s multiple comparisons test), but their T_c_ was comparable to that of WT or HET littermate controls when the ambient temperature was held at 37 °C (Fig. 4*E*, *P* > 0.05, one-way ANOVA). The slopes of T_c_ change with ambient temperature manipulation also differed between pups with TMEM16C eleminated from *Ptgds*-expressing cells and littermate control pups (Fig. 4 *H*–*J*, *P* < 0.01, one-way ANOVA followed by Tukey’s multiple comparisons test). These findings reveal a highly significant deficiency of body temperature regulation of these cKO mouse pups as compared with littermate controls of the same age.

In summary, the poor thermoregulation of cKO mouse pups with TMEM16C elimination from *Ptgds*-expressing cells but not from the majority of hippocampal and cortical neurons or sensory neurons, taken together with their normal susceptibility to chemoconvulsant-induced seizures, suggest that their greater susceptibility to hyperthermia-induced seizures likely derives from their deficiency in homeostatic thermoregulation.

## Discussion

FSs, the most prevalent convulsion in young children (2–4), warrant attention in both the clinics and the laboratories (32, 33). The risk is greater for children with family history of FSs; several genetic risk factors have been identified (15–17). However, it remains an intriguing question as to how seizures are induced by fever (34). It has been proposed that the risk for FSs could be raised by either a rapid increase of T_c_ or by altering the T_c_ threshold for seizure induction (6, 7). To date, there are limited clinical data in support of either hypothesis. Using rodent models with TMEM16C eliminated from all central neurons or a subset of central neurons, we explored seizure genesis induced by hyperthermia. We found that *Tmem16c* KO rat pups and *Tmem16c* cKO mouse pups were significantly more susceptible to hyperthermia-induced seizure (Figs. 1*D* and 3 *A*–*D* and *SI Appendix*, Fig. S3 *A*–*D*), and the seizure susceptibility was associated with a rapid rise of T_c_ (Figs. 1 *B*, *F*, and *G* and 3 *E* and *F* and *SI Appendix*, Fig. S3 *E* and *F*).

*TMEM16C* has been implicated as a genetic risk factor for FSs in a GWAS study (15). Whereas the presence of the genetic variant within the first intron leaves opens the question as to how *TMEM16C* may be involved in febrile seizures, mutant analyses of rodents lacking *Tmem16c* function raise the prospect that both hyperexcitability of central neurons and compromised thermoregulation may play a role. Our previous electrophysiological studies of *Tmem16c* KO rat pups support the notion that hippocampal neuronal hyperexcitability could contribute to seizure susceptibility (15). In addition, our surprise finding that loss of TMEM16C function results in a drastic reduction of warm-sensitive POA neurons (15), a neuronal population that plays a pivotal role in thermoregulation (25–29), has raised the intriguing question about TMEM16C involvement in thermoregulation. It is conceivable that TMEM16C function in either the hippocampus or POA, or the combination of both, could contribute to FS susceptibility. To look into the cellular contribution of TMEM16C to FS susceptibility, we generated mice with floxed alleles of *Tmem16c* and crossed them with mice that express Cre recombinase driven by the *Ptgds* promoter (31). PGDS-Cre marks the temperature-sensitive POA neurons that control thermoregulation (30), and it was expressed in a small fraction of the *Tmem16c*-expressing neurons in the cortex and hippocampus (*SI Appendix*, Fig. S2) as well as sensory neurons in the DRG (Fig. 2). These mutant mouse pups with TMEM16C removed from *Ptgds*-expressing cells were more susceptible to hyperthermia-induced seizure, and the seizure onset was associated with a more rapid rise in T_c_ (Fig. 3), thus implicating poor thermoregulation in hyperthermia-induced seizure susceptibility.

Whereas analyses primarily based on anatomical data and onset times of chemical markers indicate that the rodent age of P10 to P11 is equivalent to the third trimester of human gestation (35), different brain structures and network functions have different developmental time courses in various mammalian species, and hyperthermia-induced seizures occur mainly during the second postnatal week in rodents (24, 36). Our observations are consistent with previous report that the seizures are induced most reliably and are most stereotyped in P10 to P11 pups (23). Behaviorally, rodents at the age of P10 to P11 are more equivalent to human toddlers at the age of 1.5 y old. Hence, thermoregulation of P10 to P11 rodent pups, albeit still immature, is of relevance to the study of hyperthermia-induced seizure as a model of febrile seizures. As the expression of *Ptgds* is not limited to temperature-sensitive POA neurons in P11 pups (*SI Appendix*, Fig. S2), we cannot exclude the possibility that TMEM16C expressed in other brain regions also contributes to hyperthermia-induced seizures. Unfortunately, it is difficult to conduct brain region-specific knockout or knockdown of TMEM16C in mouse pups at such a young age. It is important to point out, however, that TMEM16C elimination from *Ptgds-*expressing cells did not alter the chemoconvulsant-induced seizure susceptibility (Fig. 3*H*). It is also notable that the great majority of DRG neurons with *Tmem16c* mRNA expression did not express *Ptgds* mRNA (Fig. 2). Those findings support the notion that it is the PGDS-Cre-mediated knockout of TMEM16C from a subset of neurons in the POA that is critical to hyperthermia-induced seizures.

Our observation that *Tmem16c* cKO mouse pups exhibited a greater rate of T_c_ change upon exposure to different ambient temperatures (Fig. 4 *F* and *G*) argues strongly that these mutants are deficient in thermoregulation for maintaining their T_c_, most likely owing to the loss of TMEM16C function in temperature-sensitive POA neurons that mediate bidirectional regulation of body temperature (30). Remarkably, when the ambient temperature was held at 22 °C, the T_c_ of mouse pups with TMEM16C removed from *Ptgds*-expressing cells was 3 °C lower than that of their littermate WT control (Fig. 4). To our knowledge, this is the largest deviation of T_c_ documented in a mutant animal. This is particularly significant given that the T_c_ is normally maintained within 0.5 °C of the set point, and deviation by 0.2 °C above or below the set point would trigger reflex activities for heat loss or heat gain, respectively (37). Whereas these *Tmem16c* cKO pups are significantly impaired, as compared to sibling controls at the same age, in their ability to maintain body temperature when held at room temperature (Fig. 4), it is important to note that thermoregulation of P11 pups is immature and these cKO mutant pups displayed normal T_c_ at elevated ambient temperature that would be attainable as pups huddled in the nest with their dam. In this setting, there is less demand for thermoregulation to maintain the body temperature near the set point—a condition that may have helped *Tmem16c* cKO mouse pups to survive. Importantly, heat exposure resulted in accelerated T_c_ rise of mutant pups with TMEM16C removed from the entire brain or *Ptgds*-expressing cells, which may enhance their susceptibility for hyperthermia-induced seizure (*SI Appendix*, Fig. S3 and Fig. 3).

How TMEM16C might contribute to the temperature sensitivity of POA neurons is an intriguing open question. In nociceptive DRG neurons, TMEM16C is an auxiliary subunit of the Slack Na^+^-activated K^+^ channel (20). In hippocampal neurons lacking TMEM16C, the hyperexcitability likely arises from a deficit in K^+^ channel function (15). It will be of interest to identify the channel and/or other proteins associated with TMEM16C in temperature-sensitive POA neurons, so as to enable the assessment of their contribution to the temperature-dependent changes of action potential firing in these hypothalamic neurons in future studies.

It remains an open question as to how eliminating TMEM16C from *Ptgds*-expressing POA neurons may lead to poor thermoregulation. It is conceivable that TMEM16C ablation in heat-sensitive POA neurons leads to hyperexcitability, which further activates downstream neurons to reduce body temperature. It is also possible that this genetic manipulation renders *Ptgds*-expressing POA neurons less sensitive to temperature changes in this brain region, given that we found no alteration of PGDS expression in the POA of these cKO mice. It will be of interest to pursue studies of *Ptgds*-expressing POA neurons with or without TMEM16C or proteins associated with TMEM16C, to address this intriguing open question.

In summary, our findings reveal that TMEM16C is critical to the homeostatic thermoregulation in protecting animals from hyperthermia-induced seizure. Consistent with this perspective, removal of TMEM16C from *Ptgds*-expressing cells, but not from the majority of hippocampal and cortical neurons in the central nervous system as well as sensory neurons in the DRG, renders the mutant pups more susceptible to hyperthermia-induced seizure as a model of febrile seizures. With recent studies elucidating a range of circuitries and mechanisms for thermoregulation (8, 9, 38–48), it will be of interest to identify those elements involved in thermoregulation that play a role in the genesis of febrile seizures.

## Materials and Methods

### Experimental Rodent Models.

The transgenic rat line of *Tmem16c* KO was generated by DNA transposon insertional mutagenesis (20). Rat pups at the age of P10, the corresponding age of young children with febrile seizures (24, 36), were used in hyperthermia-induced seizure tests. They were generated by breeding pairs of heterozygous (^+/−^) mice.

The *Tmem16c* Flox/+ mice were engineered with a recombineering method for targeting loxP sites as previously described (49) using plasmids (PL451 and PL452) and recombinogenic bacteria (SW102 and SW106) from the National Cancer Institute at Frederick, MD. The targeting vector was cloned from BAC RP23-371L6 (Children’s Hospital Oakland Research Institute). A frt-neo-frt-loxP cassette was inserted in the 5′ intronic region of exon 19 and a loxP site was inserted in the 3′ intronic region of exon 20 in the targeting vector. The linearized targeting vector was electroporated into C57BL6/129SvJ-5 F1 embryonic stem (ES) cells (Applied StemCell). Successfully targeted ES cells were selected with G418 and Gancyclovir and confirmed by PCR. Correctly targeted ES cells were then injected into blastocysts and germline transmission was confirmed by PCR. The resulting mouse was then bred with β-actin-Flp mouse to delete the neomycin cassette.

The transgenic mouse line B6.Cg-Tg(Nes-cre)1Kln/J (Nestin-Cre, MGI: 2176173) was imported from The Jackson Laboratory; the transgenic mouse line FVB.Cg-Ptgds < tm1(cre)Gvn>/GvnRbrc (PGDS-Cre, MGI:5051626) (31) was imported from RIKEN BRC, Japan. They were crossed with *Tmem16c* Flox/+ mice to generate the cKO line of TMEM16C. Mouse pups at the age of P11, the corresponding age of young children with febrile seizures (24, 36), were used in most of the experiments. They were generated by breeding pairs of Cre^−^, *Tmem16c* flox/flox ♀ crossed with Cre^+^, and *Tmem16c* flox/+ ♂.

All rats and mice were maintained under a 12:12 h light/dark schedule, and they were allowed to receive food and water ad libitum. Both males and females, which displayed similar phenotypes, were included in this study. All protocols were approved by the Institutional Animal Care and Use Committee at the University of California, San Francisco, in full compliance with NIH guidelines for humane treatment of animals.

### Hyperthermia-Induced Seizures.

The procedure was adapted from established protocols in rat and mouse pups (23, 24). The rat pups were tested at the age of P10, while the mouse pups were tested at the age of P11. Only one pup was tested at a time. Baseline core (rectal) temperature of each animal was measured by a thermistor probe (RET-4, PhysiTemp). The thermistor was inserted into the rectum and taped to the tail with the pup held unanesthetized in hand. Once the rectal temperature probe was set, the pup was released and allowed to run free in a 2.5-L crystallizing dish, with the bottom covered with a heat-resistant carpet. The body temperature of each tested animal and the ambient temperature in the chamber (measured with probe MT-D, PhysiTemp) were monitored and recorded at the sampling rate of 1 s with a data acquisition system (THERMES USB, PhysiTemp).

After a 2-min recording at rest (room temperature ∼22 °C), heated air was blown into the chamber using a low noise hair dryer (Panasonic). Airflow was adjusted to maintain the ambient temperature in the chamber at 45 to 50 °C. The core temperature of the tested animal was monitored and recorded while its behavior was observed and videotaped. According to the published protocol, rat pups subjected to hyperthermia exhibited stereotypical seizure behaviors of sudden stopped movement, tonic flexion, and hindlimb chewing; while mouse pups subjected to hyperthermia exhibited stereotypical seizure behaviors in four progressive phases: I) hyperactivity, jumping, or rearing; II) sudden immobility, ataxia, or jerky gait; III) running in circles, whole-body shaking, contractions of hind- and forelimbs with reduced consciousness; and IV) tonic convulsions with loss of consciousness. Immediately at the onset of seizures (phase IV for mouse), each tested pup was removed to a cool surface, and the seizure behavior stopped. All the tested animals were killed after the experiment. Before the next round of tests, the hyperthermia chamber was rinsed and dried for hygiene, so that the temperature of the chamber floor remained at ∼22 °C.

In determining the latency to the onset of seizures, the behavior video of each animal was reviewed and evaluated by a researcher blind to the genotype of the tested animal. To be consistent, all the video was scored by the same researcher in a short period (∼3 d). Meanwhile, the recording of rectal temperature of the tested animal was aligned to the behavior video, so as to obtain the threshold temperature at each phase of a seizure. In addition, the rectal temperature of the tested animal at rest was obtained from the last 10 s before exposure to hyperthermia; and the slope of temperature rise during hyperthermia was calculated by linear regression from the temperature recording trace.

The rest and threshold temperatures, latency to each phase of seizure, and the slope of temperature rise were categorized by the three genotypes of the tested animals. One-way ANOVA followed by Tukey’s multiple comparisons test were applied to determine significant difference.

### Tracking the Changes of Body Temperature of Mouse Pups in a Temperature-Controlled Chamber.

Mouse pups at the age of P11 were challenged with elevated ambient temperature at 30 and 37 °C, which is higher than room temperature in the mouse colony, but not too high to induce seizures. Their body temperature was recorded and monitored by the same set-up as described above for hyperthermia-induced seizure tests, except that the pups ran free in a regular mouse cage with bedding, instead of a crystallizing dish, before they were transferred to a rodent incubator with programmable ambient temperature control (RIS33SD, Powers Scientific), six cages at a time, with only one tested animal in each cage.

In the test, the rectal temperature probe was inserted into the animal before it was released and allowed to run free in a clean cage in the incubator, with the ambient temperature set at 22 °C. The habituation took 5 to 10 min before the recording started. The ambient temperature in the incubator was 1) held at 22 °C for 30 min; then 2) raised to 30 °C and held for 30 min; 3) further raised to 37 °C and held for another 30 min; and 4) set back to 22 °C, and the recovery period lasted for 60 min. Note that the ramp time of incubator temperature changes was ∼1 °C/min and times listed above were the durations once the ambient temperature set point was reached; the entire temperature cycle was controlled by the program of the incubator, which minimized the variance in temperature manipulation from test to test. The core body temperature of pups was recorded during the entire process at the sampling rate of 1 s. Tested animals were killed immediately after the procedure.

The pup body temperature at 22, 30, and 37 °C was obtained from the last 5 min of each episode (before temperature change); the slope of temperature drop at 22 °C, the slope of temperature rise from 22 to 30 °C, and the slope of temperature rise from 30 to 37 °C were calculated by linear regression from the recording traces. The measured data were categorized by the three genotypes of the tested animals. One-way ANOVA followed by Tukey’s multiple comparisons test were applied to determine significant difference.

### Chemoconvulsant-Induced Seizures in Mouse Pups.

The procedure was adapted from established protocols in mouse epilepsy studies (50). Mouse pups at the age of P11 were used for this experiment. Intraperitoneal injection of kainic acid (5 mg/kg) induced seizures in 100% of the animals tested. The behaviors, including automatisms and a catatonic posture that often progressed to myoclonic twitching of the head, forelimbs, and rear limbs, were observed to determine the latency of seizure onset, by using the same criteria as for hyperthermia-induced seizures. All the tested animals were killed by the end of the experiments.

### In Situ Hybridization Using RNAscope.

P11 mice were perfused with 10 mM phosphate buffered saline (PBS) in diethyl pyrocarbonate (DEPC) water. Fresh frozen brains were sectioned at 14 μm at −20 °C. RNAscope was performed following the ACDBio RNAscope Fluorescent Multiplex Reagent Kit User Manual (320293-UM). In brief, the specimen was incubated with 1) Hybridize Probes for *Tmem16c* and *Ptgds* at 40 °C for 2 h; 2) Hybridize Amp 1-FL at 40 °C for 30 min; 3) Hybridize Amp 2-FL at 40 °C for 15 min; 4) Hybridize Amp 3-FL at 40 °C for 30 min; and 5) Hybridize Amp 4-FL at 40 °C for 15 min, with three 2-min washings at room temperature between each step. After incubating with DAPI for 30 s at room temperature, the specimen was mounted onto glass slides and coated with Fluoroshield (MilliporeSigma). The specimen was imaged with a confocal microscope (Leica SP5) after drying.

### Immunostaining.

The immunostaining protocol used in this study is identical to that in our previous study (30). Briefly, after perfusion of mice with 4% paraformaldehyde (PFA) in PBS through the left ventricle and postfixing of mouse brains in 4% PFA overnight, we used a vibrating blade microtome (Leica) to generate 40-µm slices, which were washed in Tris-buffered saline (TBS, 50 mM Tris, 150 mM NaCl, pH 7.4) three times and processed for heat-induced epitope retrieval in 10 mM Na-citrate with 0.05% Tween 20, pH 6.0, at 80 °C, for 30 min. After washing three times in TBS with 0.3% Triton 100 (TBS-T) and then in the blocking buffer (TBS with 0.3% Triton 100, 10% normal donkey serum, and 2% bovine serum albumin) at room temperature for 2 h, the brain slices were incubated with primary antibodies against L-PGDS (mouse anti PGD_2_ synthase (F-7), 1:200, Santa Cruz, sc-390717, RRID:AB_2800545) at 4 °C overnight, washed in TBS-T three times, and then incubated with secondary antibodies of donkey anti-mouse IgG conjugated with RRX (1:1,000, Jackson ImmunoResearch) plus DAPI (1:5,000, MilliporeSigma), followed by three washes in TBS. Finally, the brain slices were mounted onto glass slides and coated with Fluoroshield (MilliporeSigma) for imaging with a confocal microscope (Leica SP5).

## Supplementary Material

Supplementary File

## Data Availability

All study data are included in the article and/or supporting information.
