# Supplementary material for: TMEM16C is involved in thermoregulation and protects rodent pups from febrile seizures
Source: Proc Natl Acad Sci U S A. 2021 May 10;118(20):e2023342118. doi: 10.1073/pnas.2023342118 (PMC8157992; doi:10.1073/pnas.2023342118)
Supplement: Supplementary File [file pnas.2023342118.sapp.pdf]

PNAS
www.pnas.org

Supplementary Information for

TMEM16C is Involved in Thermoregulation and Protects Rodent Pups from Febrile Seizures

Tongfei A. Wang[a,1], Chao Chen[a], Fen Huang[a,2], Shengjie Feng[a], Jason Tien[a,3], João M. Braz[b], Allan I. Basbaum[b], Yuh Nung Jan[a,c], Lily Yeh Jan[a,c,*]

*Lily Yeh Jan
Email: Lily.Jan@ucsf.edu

This PDF file includes:

Figures S1 to Sx (not allowed for Brief Reports)

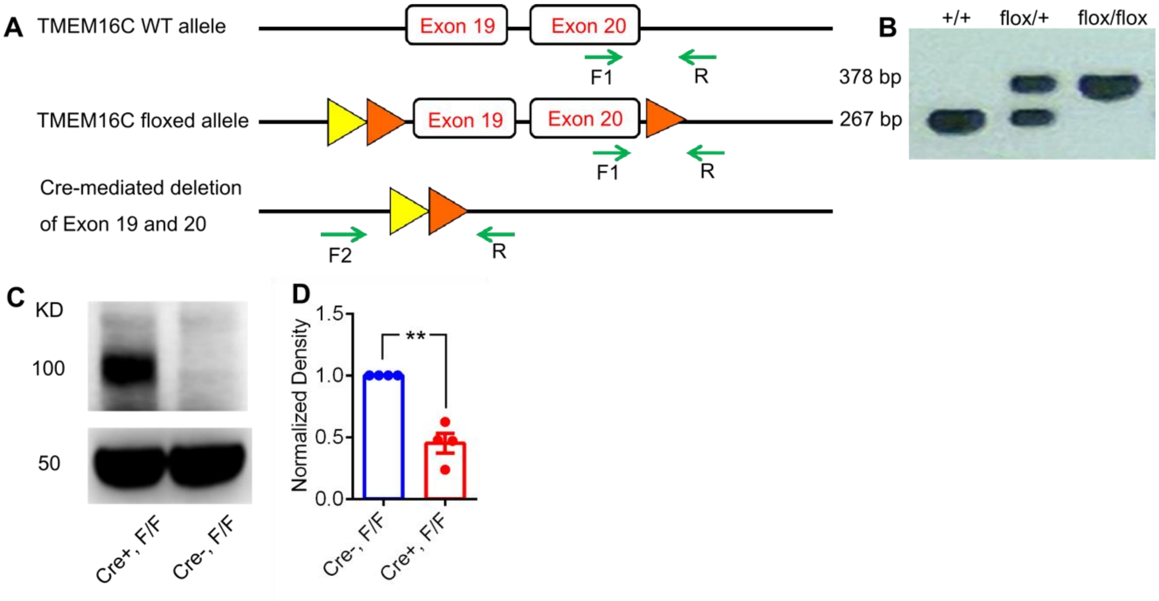

Fig. S1. Mouse line with a floxed TMEM16C allele.

(*A*) Design for the conditional knockout of TMEM16C. (*B*) Genotyping sample gel. (*C* and *D*) Western blot detection of TMEM16C expression in the whole brains of the mice with or without Nextin-Cre expression, and homozygous floxed alleles of TMEM16C; n = 4; $p < 0.01$, Student's T-test; data present in mean ± s.e.m.

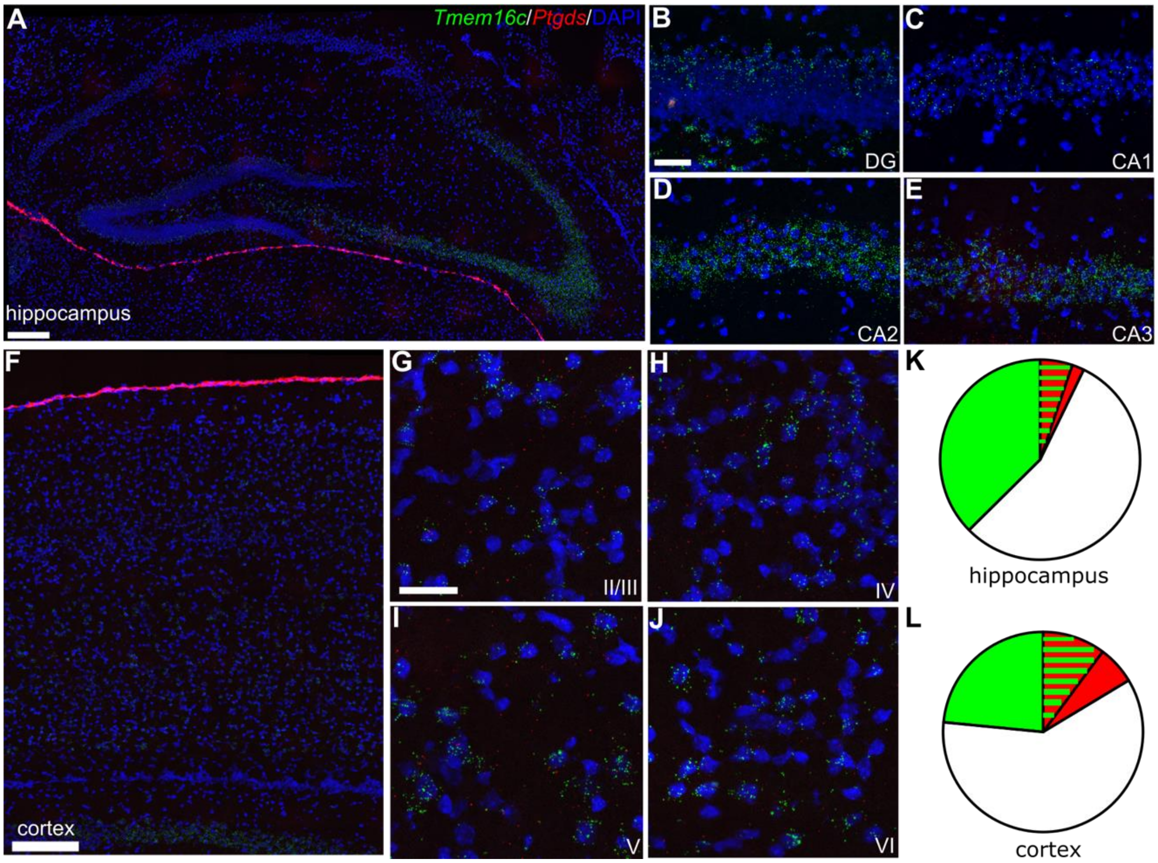

Fig. S2. TMEM16C expression in some of the *Ptgds*-expressing neurons in the hippocampus and cortex.

RNAscope of *Tmem16c* (green), *Ptgds* (red), and DAPI (blue) in hippocampus (*A*–*E* and *K*) and cerebral cortex (*F*–*J* and *L*) from mouse pups of P11 (3 replicates) reveals that a subset of TMEM16C expressing neurons also express *Ptgds*; scale bar: 200 µm in *A* and *H*; 50 µm in *B* and *G*.

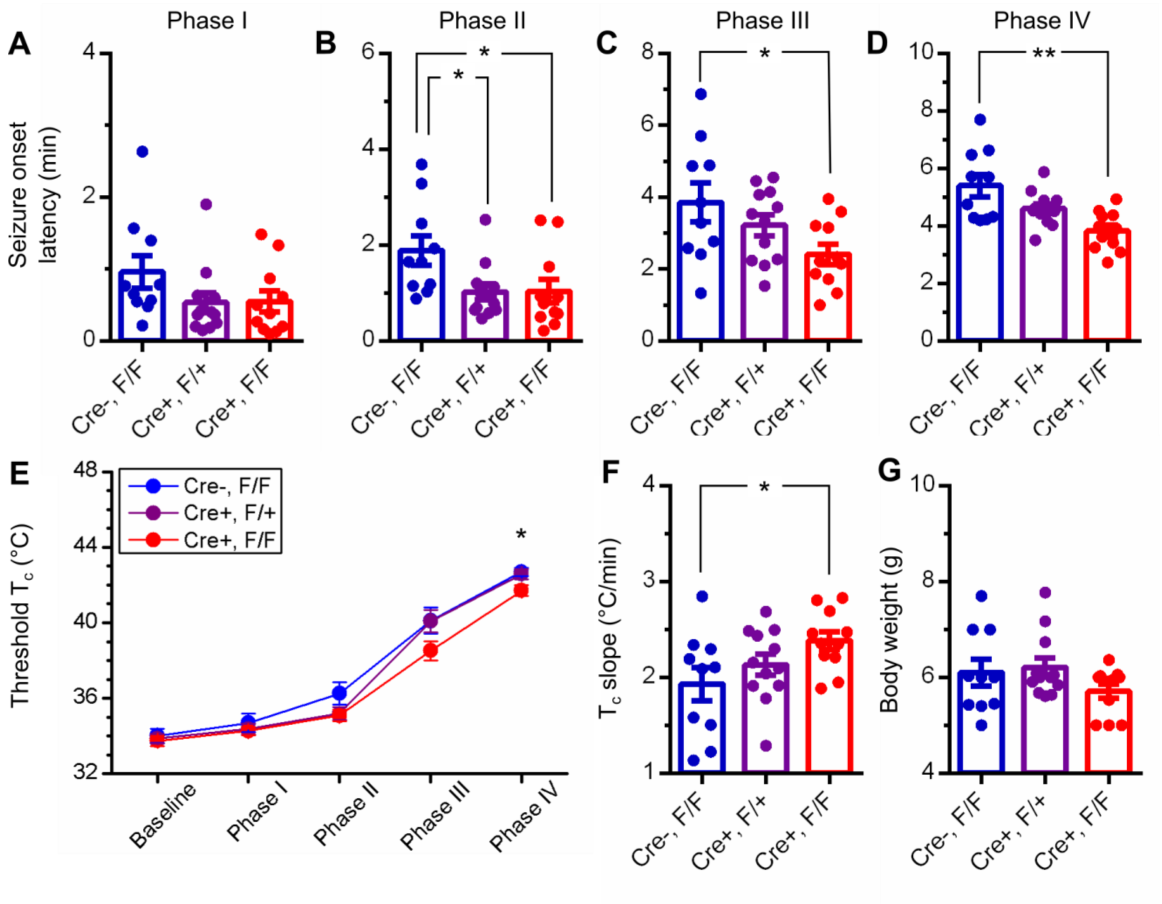

Fig. S3. Mouse pups (P11) with TMEM16C removed from the brain via Nestin-Cre are more susceptible to hyperthermia-induced seizure.

(*A*–*D*) Latency for mouse pups (blue, Cre-, flox/flox; purple, Cre+, flox/+; red, Cre+, flox/flox; n = 12 in each genotype) to enter the seizure phases I (*A*, hyperactivity, jumping or rearing), II (*B*, sudden immobility, ataxia or jerky gait, $p < 0.05$, One-Way ANOVA), III (*C*, circling running, whole-body shaking, contractions of hind- and forelimbs with reduced consciousness, $p < 0.05$, One-Way ANOVA), and IV (*D*, tonic convulsions with loss of consciousness, $p < 0.01$, One-Way ANOVA); **, $p < 0.01$; *, $p < 0.05$, Tukey's multiple comparisons test; data present in mean ± s.e.m. (*E*) Rectal temperature of mouse pups at each phase of seizure; $p < 0.05$, One-Way ANOVA; *, $p < 0.05$, Tukey's multiple comparisons test in phase IV; data present in mean ± s.e.m. (*F* and *G*) Rectal temperature increase rate (*F*, $p < 0.05$, One-Way ANOVA; *, $p < 0.05$, Tukey's multiple comparisons test) and body weight (*G*, $p > 0.05$, One-Way ANOVA) of mouse pups in hyperthermia-induced seizure test; data present in mean ± s.e.m.

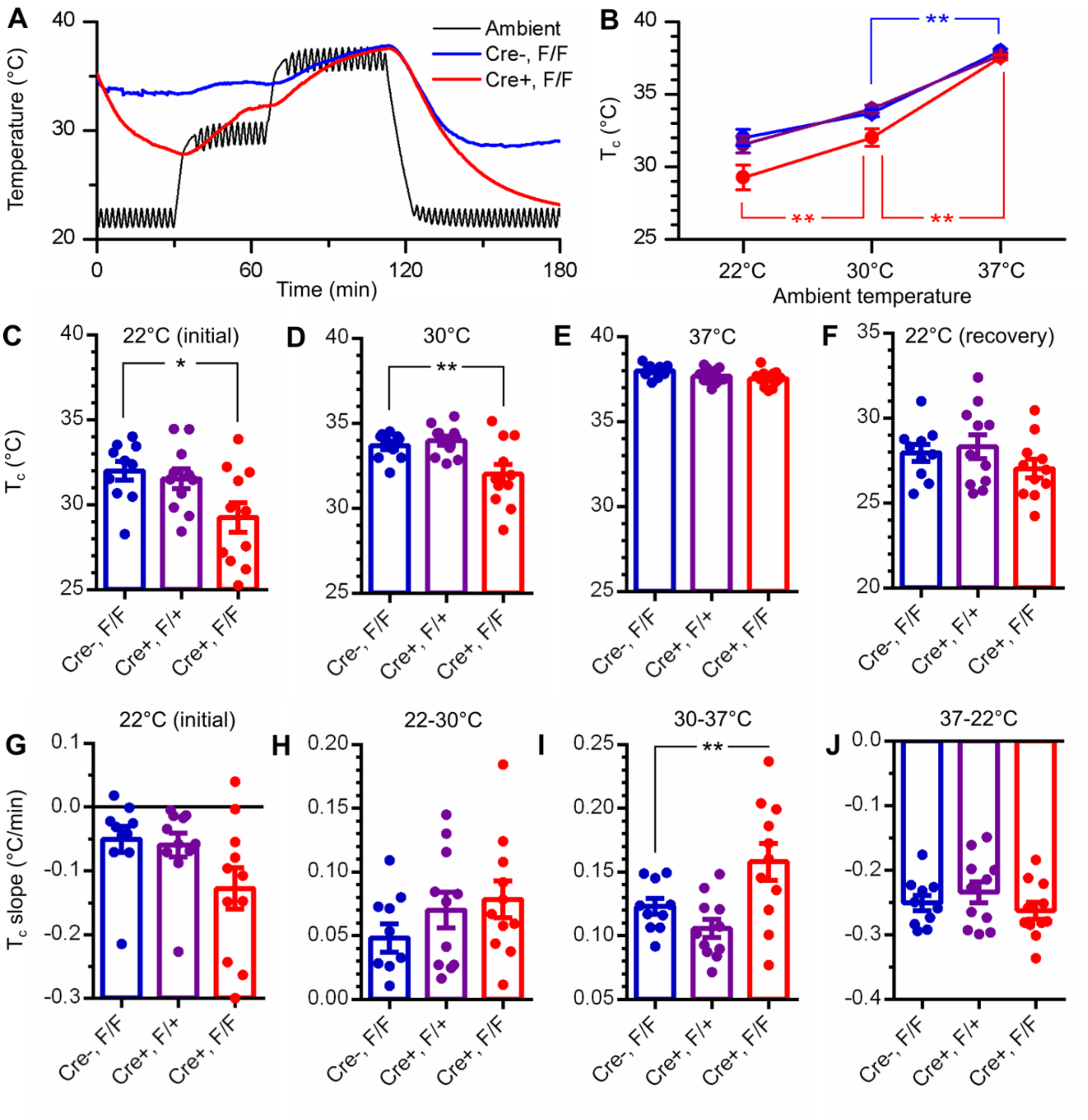

Fig. S4. Mouse pups (P11) with TMEM16C removed from the brain via Nestin-Cre exhibited abnormal body temperature.

(*A*) Sample traces of rectal temperature recording in mouse pups with or without TMEM16C in *Ptgds*-expressing neurons (blue, Cre-, flox/flox, n = 12; red, Cre+, flox/flox, n = 11) in response to elevated ambient temperature (black). (*B–F*) Summary of animals' rectal temperature in test ambient temperatures of 22°C (*C*, at rest, $p < 0.05$, One-Way ANOVA), 30°C (*D*, $p < 0.01$, One-Way ANOVA), 37°C (*E*), and 22°C (*F*, recovery from heat exposure); purple, Cre+, flox/+, n = 12; **, $p < 0.01$; *, $p < 0.05$, Tukey's multiple comparisons test; data present in mean ± s.e.m. (*G–J*)

Animals' rectal temperature change slopes in ambient temperature of 22°C at rest (*G*), from 22°C to 30°C (*H*), from 30°C to 37°C (*I*, $p < 0.01$, One-Way ANOVA), and from 37°C to 22°C (*J*, recovery from heat exposure); **, $p < 0.01$; *, $p < 0.05$, Tukey's multiple comparisons test; data present in mean ± s.e.m.
